# Atypical Duplex appendix arising from the ascending colon: a case report

**DOI:** 10.1186/s13256-023-04259-4

**Published:** 2024-03-29

**Authors:** Ahmed Taha Elsherbini, Mohamed A. Atta, Sahar Elshahat, Mohamed H. Emara

**Affiliations:** 1 Surgery Department, Faculty of Medicine, Mansoura University, Mansoura, 33516 Egypt; 2 Surgery Department, Alyousif Hospital, Al-Khobar, Saudi Arabia; 3 Radiology Department, Alyousif Hospital, Al-Khobar, Saudi Arabia; 4 Clinical Pathology Department, Faculty of Medicine, Al-Azhar University, Cairo, Egypt; 5 Clinical Pathology Department, Alyousif Hospital, Al-Khobar, Saudi Arabia; 6 Hepatology, Gastroenterology and Infectious Diseases Department, Faculty of Medicine, Kafrelsheikh University, Kafr Elshikh, 33516 Egypt; 7 Medicine Department, Alyousif Hospital, Al-Khobar, Saudi Arabia

**Keywords:** Duplex appendix, Appendicitis, Appendectomy, Ascending colon, Recurrent acute abdomen

## Abstract

**Background:**

Duplex or vermiform appendix refers to the presence of an appendix beside the naturally occurring one. Although, duplex appendix emerges from the caecum most of the time, yet it is encountered in other parts of the colon. Inflammation of duplex appendix may represent not only a clinical, but also a surgical dilemma, and this would be confusing further among patients who already had prior appendectomy.

**Case presentation:**

We present a case of 29-years old Egyptian male patient with history of appendectomy one and half year before presenting to the emergency department with recurrent acute abdominal pain that was linked to duplex appendicitis abnormally emerged from the mid-ascending colon. The first episode was treated conservatively considering atypical right colon diverticulitis as a potential differential diagnosis. Seven months later the patient was treated by laparoscopic appendectomy and experienced an uneventful pot-operative course.

**Conclusion:**

Duplex appendicitis, though rare, should be considered in the differential diagnosis of recurrent acute abdomen even after appendectomy.

## Introduction

The term duplex or vermiform appendix refers to the presence of an additional appendix beside the naturally occurring appendix that classically emerges from the caecum. The description of duplex/vermiform appendix is not novel [1], however it is gaining popularity in parallel with the emergence of advanced diagnostic imaging modalities [2] and the increasingly described atypical presentations focused in the literature [3]. In most cases described in the literature the extra-appendix arise from the caecum in relation to the naturally occurring one. The most commonly used classification system is the Cave-Wallbridge classification that was proposed by Wallbridge and later modified aiming to accommodate the expected atypical positions of the duplex appendix [4].

Acute appendicitis and appendectomy is the most commonly encountered non-traumatic surgical emergency performed. Consequently, the variability in appendicular position within the abdomen is likely a common cause of confusion not only for clinicians but also for surgeons. However, improved imaging modalities honed the physician's ability to diagnose this condition with high accuracy [4].

Literature evaluation of the published cases showed diverse clinical presentation, variable imaging findings and erratic anatomical positions, however, all were consistent in their message to alarm clinicians and surgeons to consider appendix duplication in the differential diagnosis of acute abdominal pain [3]. Although duplex appendix was not limited to specific age group, all ages from neonatal period to elderly have been affected, yet young adults are mostly manifested [3, 5].

## Case presentation

**Fig. 1:**
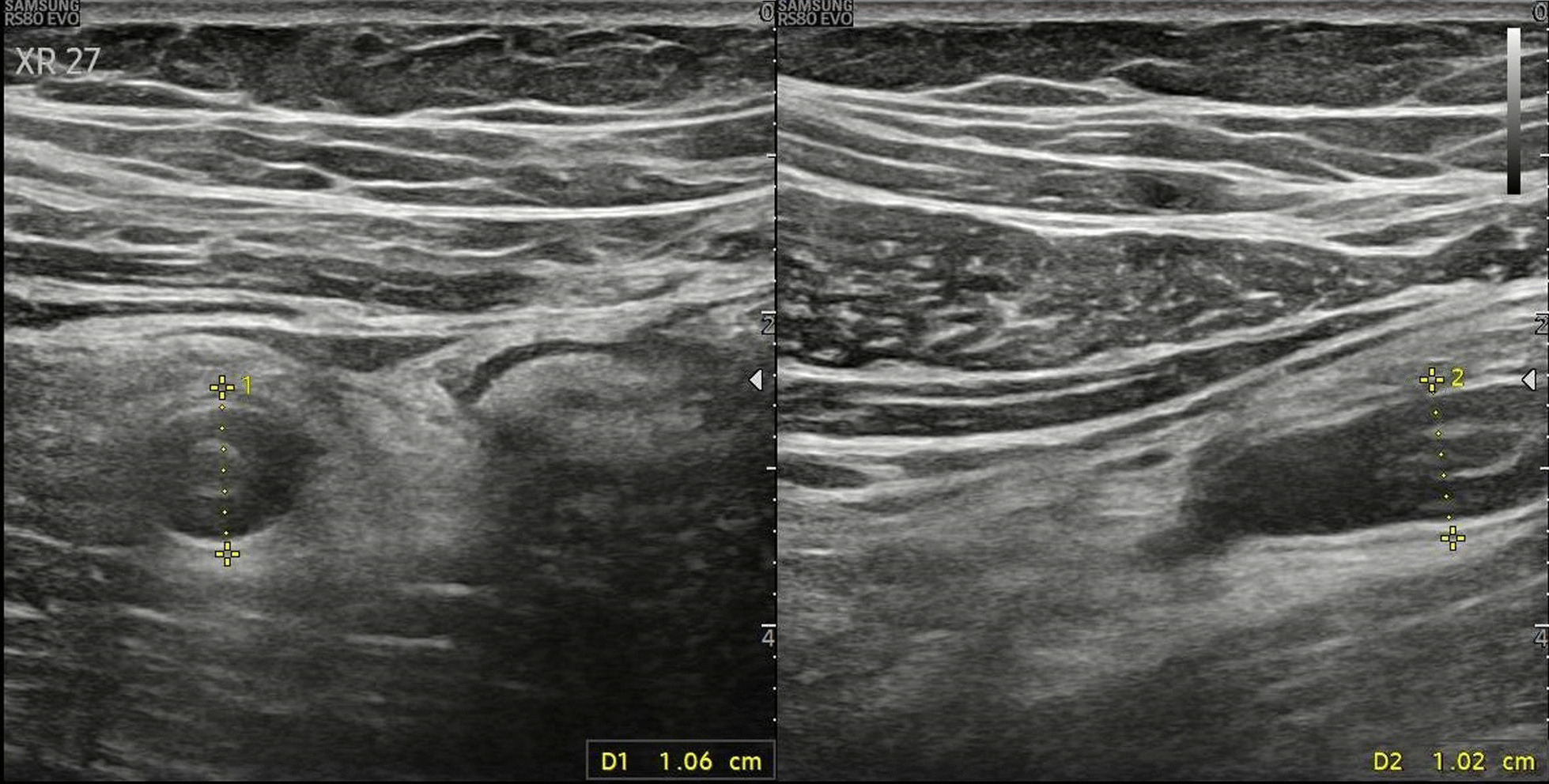
Abdominal ultrasonography showing a well-defined blind ended tubular non compressible structure seen at the upper limits of the right iliac fossa measuring about 3 cm in length and 1 cm in diameter

A 29-year old Egyptian male patient presented to Emergency room (ER) with acute, severe, colicky, steady increasing, right lumbar and right iliac fossa pain associated with nausea and vomiting. On examination he had Blood pressure (BP) 143/74 mmHg, T37.4 °C, Pulse 77/minute, and Oxygen (O2) saturation 99%. Local abdominal examination showed a small right iliac fossa (RIF) scar of previous appendectomy, local tenderness with rebound. The patient gave history of appendectomy 1 ½ years earlier and was evaluated in the ER as a case of acute abdomen with probability of stump appendicitis. To figure out a final diagnosis a complete work up was done. Laboratory results showed high CRP (18 mg/dl), ESR 5, creatinine 1.4, white blood cell count of 7.2 × 10^3^/mm^3^ (81.6% neutrophils), hemoglobin 14.6 gm%, platelets 243 × 10^3^/mm^3^, and unremarkable urine analysis. Abdominal ultrasonography (US) described a well-defined blind ended tubular non compressible structure seen at the upper limits of the right iliac fossa measuring about3 cm in length and 1 cm in diameter associated with inflammatory changes at the cecum (Fig. 1), increased echogenicity of the surrounding omental fat and rim of free fluid around raising the possibility of stump appendicitis for computed tomography (CT) confirmation.

**Fig. 2:**
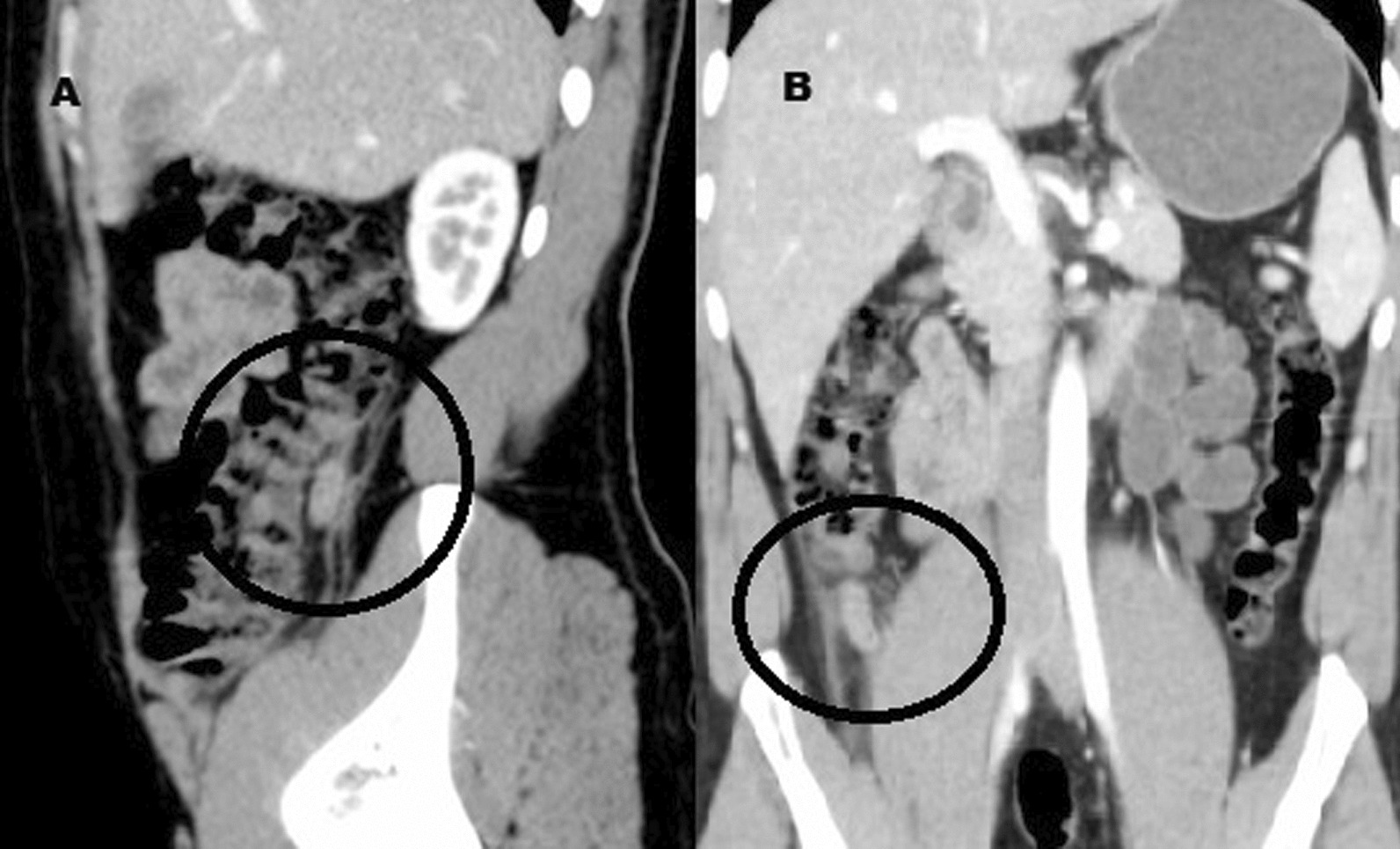
CT abdomen showing inflamed blind ended loop (circle) emerging from the posterior aspect of the middle part of the right colon (sagittal section; **A**) at the right lumber region (at a distance measuring about 3.5 cm distal to the inferior surface of the liver and about 7 cm proximal to the ileo-cecal junction, coronal section; **B**) measuring about 1 cm at maximum diameter and about 5 cm in length

CT abdomen done (Fig. 2) and showed an inflamed blind ended loop emerging from the posterior aspect of the middle part of the right colon at the right lumbar region (at a distance measuring about 3.5 cm distal to the inferior surface of the liver and about 7 cm proximal to the ileo-cecal junction) measuring about 1 cm at maximum diameter and about 5 cm in length associated with stranding of the surrounding fat planes and minimal amount of fluid collection around (inflammatory changes). Multiple mildly enlarged reactive mesenteric lymph nodes (LNs) were also seen. The patient was admitted and treated conservatively by injection levofloxacin and metronidazole for the possible differential diagnosis of acute diverticulitis.

**Fig. 3:**
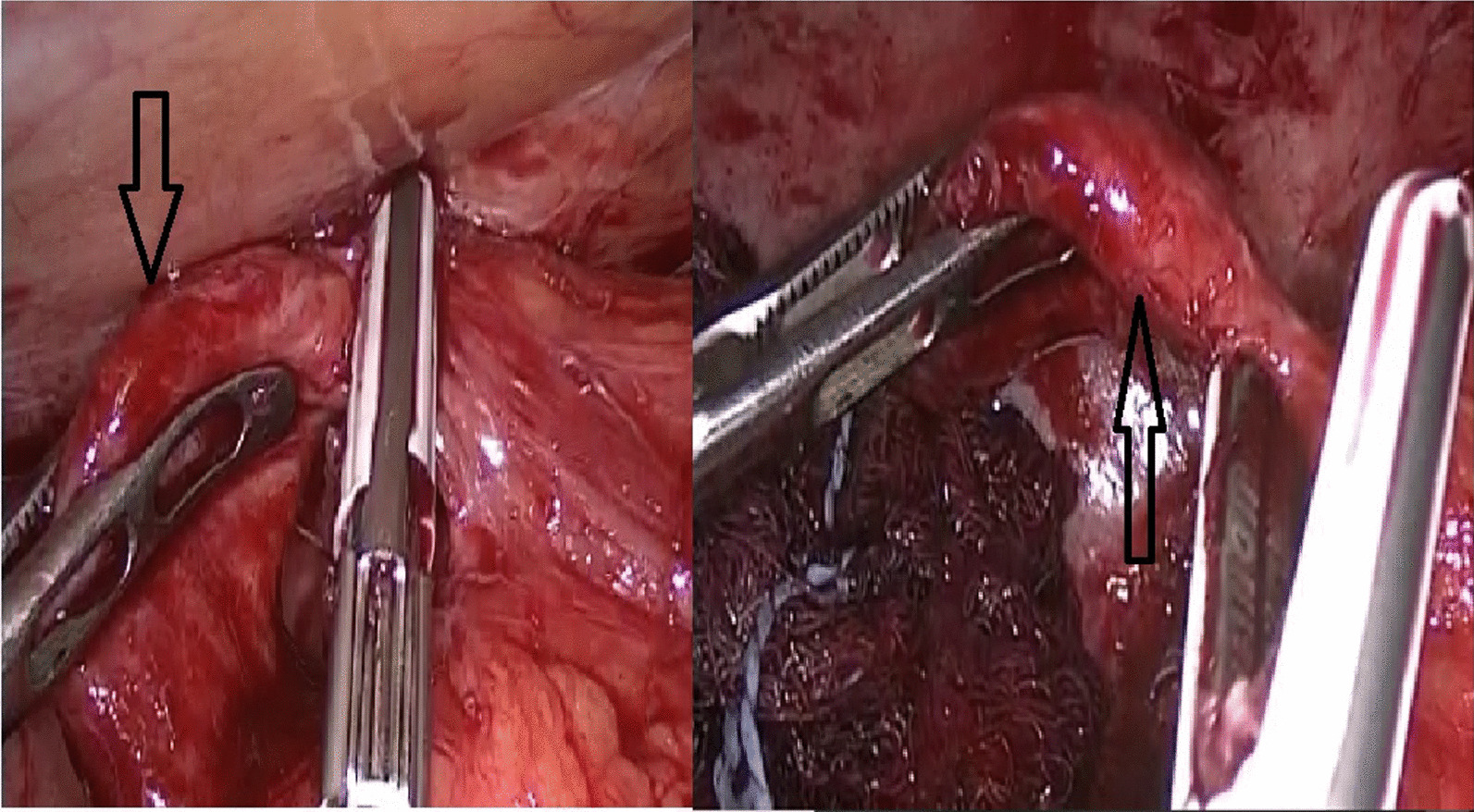
Laparoscopy; showed the duplex appendix (arrows) while dissection before excision

The patient was discharged after 3-days and followed in the out patient department (OPD). The patient during OPD follow up visits offered colonoscopy examination, but he refused and missed for further follow up. Seven months later the patient again presented to ER with acute, severe, colicky, right lumbar and right iliac fossa pain associated with nausea and vomiting. US showed well defined blind ended tubular non compressible structure seen at the right lumber region emerging from the right colon measuring about 3.3 cm in length and 1 cm in diameter associated with increased echogenicity of the surrounding omental fat and multiple mildly enlarged mesenteric LNs. The patient was operated on by laparoscopic appendectomy (Fig. 3) and the histopathology showed an appendix measuring 5 × 1 cm.

**Fig. 4:**
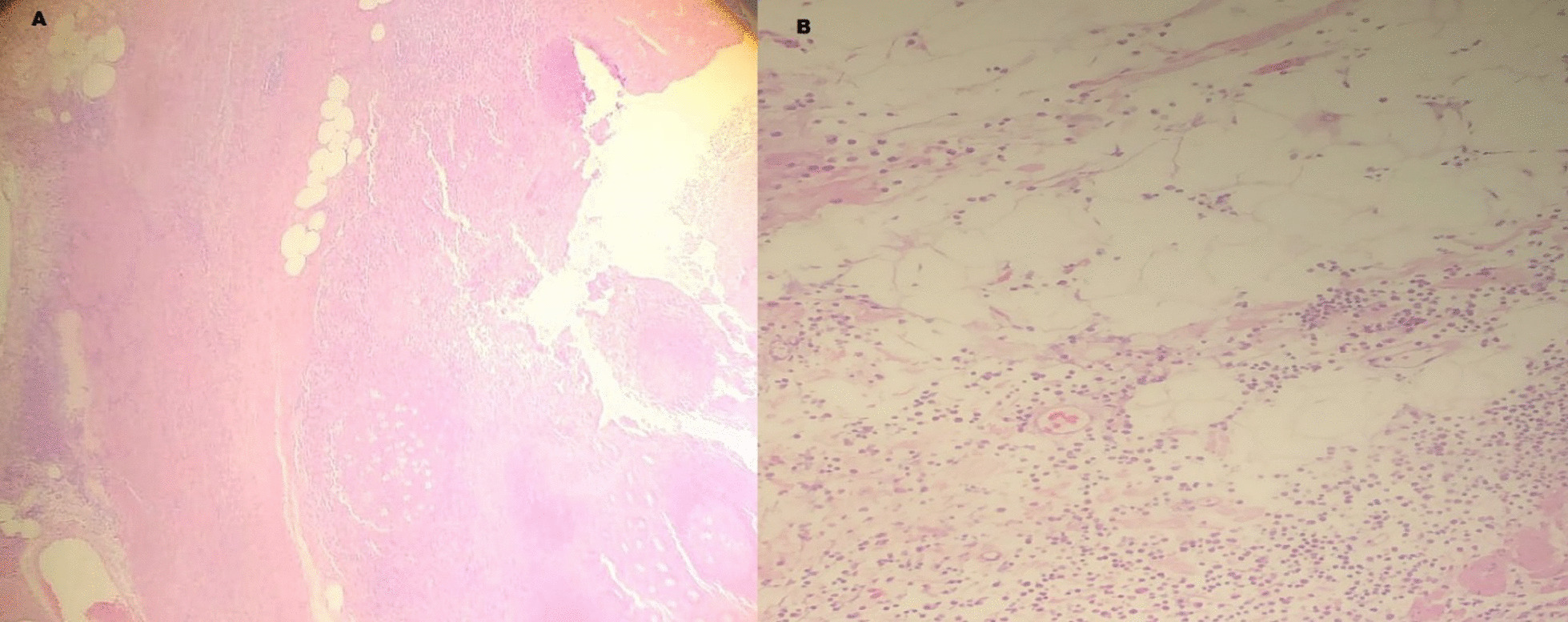
Histopathology of the removed duplex appendix showing focal ulceration of the mucosa and the mucosa is infiltrated with inflammatory cell infiltrate (**A**, **B**) associated with lymph follicle hyperplasia (**A**) that extends to the serosa (**B**)

Microscopically (Fig. 4), the sections showed appendix with focal ulceration of the mucosa associated with polymorph inflammatory cell infiltrate that extends to the serosa associated with lymph follicle hyperplasia. No granuloma or specific pathogen was identified. No evidence of malignancy was present and finally diagnosed with acute suppurative appendicitis with peri-appendicitis. The patient experienced an uneventful course and discharged from the hospital after 4-days.

## Discussion

The current case is a case of duplex appendix arising from the ascending colon and its position was confirmed by the CT examination. This atypical position was not described during the removal of the naturally occurring appendix that had been removed 1 ½ years earlier. The patient experienced 2 episodes of acute inflammation. The first episode was treated conservatively with parenteral antibiotics considering atypical diverticulitis as a potential differential diagnosis while the second episode reported 7 months later was treated by laparoscopic appendectomy.

Acute appendicitis is the most common abdominal surgical emergency [6]. However, a number of differential diagnoses should be considered [7], and the clinical examination together with the diagnostic work up should be directed to confirm the diagnosis and rule out other possible causes. Reports of appendix duplication emerged as early as 1930s-1940s [1], although the frequency is very low to around 0.004%, yet it is important to consider in the differential diagnosis of acute abdominal pain [2–6].

Duplex appendicitis as a potential cause of recurrent acute abdominal pain is infrequently reported in the literature because most cases of duplex appendix were discovered in the same hospital admission for inflammation of the natural appendix [8] or incidentally while exploring the abdomen for other conditions [9]. Most of the published cases described the duplicate appendix as non-inflamed [9], although some reports described it as inflamed in a classic fashion of the natural appendicitis [10], and sometimes on the occasion of non-inflamed natural appendix [11].

The case presented her had 2 peculiars; first and to the best of our knowledge, it is the first time for a duplicate appendix to manifest as recurrent acute appendicitis, the first episode reported 7 months before the current admission and was treated conservatively while the second episode was treated surgically. The history of appendectomy performed earlier to our patient represented a diagnostic challenge and this could easily favor a missed diagnosis of the second episode of appendicitis. Consequently, presenting with lower abdominal pain—as described in the current case 7 months before surgery- could reasonably shift differential diagnosis toward other medical conditions, *i.e.* diverticulitis of the right colon, Meckel's diverticulum, colonic cancer, gastroenteritis, acute mesenteric adenitis, intussusception, inflammatory bowel disease and other pelvic pathology thus, delaying diagnosis and appropriate treatment [11, 12], and this was actually happened with this patient when stump appendicitis was proposed initially and the likelihood of right colon diverticulitis was considered later. Vermiform appendicitis versus stump appendicitis manifested as recurrent episodes of RIF pain was recently reported by Almas *et al.* [13], however, the authors were not able to clearly demonstrate weather it was a stump appendicitis or full blown vermiform appendicitis because appendicular duplication was not reported during the index appendectomy and post-appendectomy derangements further changed the anatomy. Our case was clearly different because duplex appendix was reported emerging from the ascending colon.

Furthermore, stump appendicitis was also considered as a potential differential diagnosis during the first episode of abdominal pain encountered with the current patient. The frequency of stump appendicitis reported by an earlier huge cohort focusing cases operated by appendectomy was low and counted as 0.15% [14].

The second peculiar, is the anatomical position, here in the appendix emerged from the mid-ascending colon a position that is not covered by the widely used anatomical Cave-Wallbridge classification [4]. Even when the initial classification was enriched by modifications suggested by Biermann *et al.* [15], reporting of horseshoe and triple appendix variants, still duplex appendix arising from ascending colon and probably other atypical sites are not covered [16]. This atypical position together with history of appendectomy were the direct cause for treating the first episode of acute appendicitis in this patient conservatively considering an acute diverticulitis of elongated right colon diverticulum as a potential differential diagnosis. In fact, acute diverticulitis mimics the presentation of acute appendicitis, however the age of 29 is uncommon age for diverticulosis. We failed to categorize the current case to any of the types the Cave-Wallbridge classification and this may trigger the need to reclassify the duplex/vermiform appendix in a broader way to include more atypical sites. In fact, few earlier reports faced the same problem and failed to describe duplicate appendix to a particular type of the Cave-Wallbridge classification [3].

It is essential to obtain a histopathology report for the surgical specimen removed not only to confirm the diagnosis of acute appendicitis [11, 13], but also to rule out any differential diagnosis and complications as well. The post-operative histopathology specimen of the current case described acute suppurative appendicitis ruling out diverticulitis as a cause of the current illness.

## Conclusion

In conclusion, previous appendectomy of the naturally occurring appendix added to the momentum, and the chance for a clinician to consider an inflamed duplex appendix in this situation is unlikely and by reporting the current case we sound the alarm for clinicians especially in the ER to consider duplex appendicitis in the differential diagnosis of acute abdominal pain even after appendectomy.

## Data Availability

Available from the corresponding author upon request.
